# Neural Network of Predictive Motor Timing in the Context of Gender Differences

**DOI:** 10.1155/2016/2073454

**Published:** 2016-02-25

**Authors:** Pavel Filip, Jan Lošák, Tomáš Kašpárek, Jiří Vaníček, Martin Bareš

**Affiliations:** ^1^Central European Institute of Technology, CEITEC MU, Behavioral and Social Neuroscience Research Group, Masaryk University, 625 00 Brno, Czech Republic; ^2^First Department of Neurology, Faculty of Medicine, Masaryk University and St. Anne's University Hospital, 656 91 Brno, Czech Republic; ^3^Department of Psychiatry, Faculty of Medicine, Masaryk University and Teaching Hospital Brno, 625 00 Brno, Czech Republic; ^4^Department of Imaging Methods, Masaryk University and St. Anne's University Hospital, Brno, Czech Republic; ^5^Department of Neurology, School of Medicine, University of Minnesota, Minneapolis, MN 55455, USA

## Abstract

Time perception is an essential part of our everyday lives, in both the prospective and the retrospective domains. However, our knowledge of temporal processing is mainly limited to the networks responsible for comparing or maintaining specific intervals or frequencies. In the presented fMRI study, we sought to characterize the neural nodes engaged specifically in predictive temporal analysis, the estimation of the future position of an object with varying movement parameters, and the contingent neuroanatomical signature of differences in behavioral performance between genders. The established dominant cerebellar engagement offers novel evidence in favor of a pivotal role of this structure in predictive short-term timing, overshadowing the basal ganglia reported together with the frontal cortex as dominant in retrospective temporal processing in the subsecond spectrum. Furthermore, we discovered lower performance in this task and massively increased cerebellar activity in women compared to men, indicative of strategy differences between the genders. This promotes the view that predictive temporal computing utilizes comparable structures in the retrospective timing processes, but with a definite dominance of the cerebellum.

## 1. Introduction

Time perception is a critical element of both conscious and subconscious experience, providing synchrony and reliable representation of the surrounding environment. Despite the significance of the temporal dimension, our knowledge of the neuropsychological mechanisms underlying timing processes and computations remains relatively poor [1, 2]. Modern theories about internal time representation share the view that the processing of temporal information is mediated by a distributed network with varying engagement of its individual components depending on the task requirements [3]. However, there is substantial disagreement on the effects reported in various studies, providing no definitive anatomical or cognitive model [3–5]. Several regions, including the basal ganglia, cerebellum, posterior parietal cortex, and frontal cortex, have been implicated as relevant to interval timing; however, their precise role remains shrouded.

The traditional model describing the processing of time passage in the brain is the pacemaker-accumulator model, a straightforward solution that is surprisingly effective in explaining behavioral data [6, 7]. The pacemaker oscillates at a frequency subject to modulation by the temporal properties of sensory stimuli [8]; the pulse count is converted by the accumulator into a linear metric of time. However, recent advances point towards an alternative view offered by distributed timing models, deriving temporal information from the coincidental activation of different neural populations [1]. Although diverse, regarding the hypothesized specific neuropsychological mechanisms of time processing, this broad spectrum of models basically postulate the representation of time as ubiquitous in more networks that also encode other stimulus properties [4, 9]. Thus, temporal information may be encoded in the entire activity pattern of a neuronal mechanism, as suggested in the state-dependent network model [9, 10]. At the same time, the majority of authors agree that, in the multisecond range, the linear integration of temporal signals is a plausible mechanism for temporal coding and considers it supplemental to the second class of models arising from the inherent temporal properties of the neural networks that provides automatic timing dealing with millisecond range durations [9, 11–13]. Nonetheless, the boundary and overlap between these two systems remain to be delineated.

Of particular relevance in this context is the observation that the processing of temporal information in the millisecond range involves a different network than multisecond operations [9, 12, 14]. Following this distinction, short duration processes would be predominantly evaluated within the motor and sensory-motor network (cerebellum, premotor cortex, and sensory cortex), whereas the timing analysis in longer intervals would be associated with the striatal-prefrontal circuit [15, 16]. The suggestion that correct cerebellar function may be essential for successful temporal processing is not new [17]; its prominence in facilitating performance in time analysis has been further underscored by both clinical studies of patients with cerebellar pathology [18–22] and neuroimaging studies [15, 23–25], mostly based on paced finger tapping or temporal discrimination with the retrospective character of temporal computing (for review see [3]). However, surprisingly few studies have investigated the cerebellar role in the complex processes necessary for predictive timing—the analysis of various environmental factors in the subsecond spectrum leading to a delineation of the probable future state of the surroundings which enables preemptive action in simple, routine activities and even in life-threatening situations. Possible variations in the composition of the timing network related to gender might be of significant interest as well, as reports are available, albeit scarce, of both structural [26] and functional dissimilarities [27] between men and women in the infratentorial area. Behavioral performance differences between males and females have been well known for decades [28], with the field of numeric operations, spatial processing, and probability and data analysis dominated by men [29] and language and social skills being the realm of women [30]. Interestingly, a similar disparity has been repeatedly confirmed in complex analyses of reaction time to various stimuli revealing a significant disadvantage for women [31–34] and a differential pattern of reaction times as a function of stimuli location and character [32] that might reflect different information processing strategies. Hence, understanding the underlying neural mechanisms associated with contingent performance and functional differences in specific processes in the context of gender may offer an important insight into neurological and psychiatric diseases with strong sex biases.

Hence, the aims of the present study were twofold. We wished to establish the neuroanatomical signature of complex predictive motor timing in a well-characterized sample of healthy individuals using the fMRI blood oxygen level dependent (BOLD) signal and to contrast the performance of men to that of women.

## 2. Materials and Methods

### 2.1. Subjects

Forty healthy volunteers, 24 men (mean age 40.15; SD 15.20) and 16 women (mean age 43.85; SD 14.76), with no symptoms of neurologic diseases participated in the study. All the subjects were right-handed according to the Edinburgh Handedness Inventory [35]. All of the subjects underwent a screening to ensure a safe MRI procedure, and their anatomical MRI scans did not show any structural pathology. All participants provided their written informed consent prior to the experiment in accordance with the guidelines of the local research ethics committee. The study was approved by the institutional review board of St. Anne's University Hospital (Brno, Czech Republic).

### 2.2. Experimental Task Design

**Figure 1:**
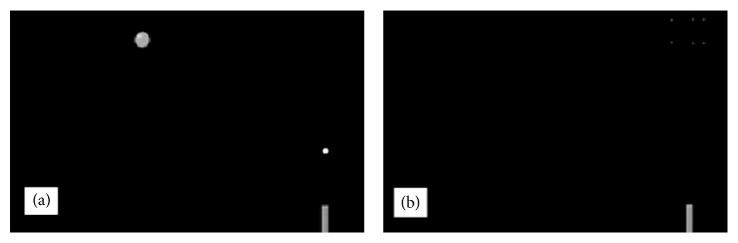
The experimental task. (a) The green ball flies from the left side of the screen to the upper right corner, the interception zone. The “gun” in the right lower corner has just fired a “projectile” travelling vertically at a constant speed to intercept the target. (b) Successful hit is associated with an “explosion” in the interception zone in the upper right corner. If the subject misses, no animation is displayed.

The participants performed the same motor-timing computer task as employed in our earlier studies [20, 22, 36, 37]. The subjects were required to press a key with the dominant hand to launch a projectile that was intended to intercept a green circular target moving from the left side of the screen toward the upper right corner (Figure 1(a)). The target (diameter 1 cm) moved to the fixed interception zone at three possible speeds (slow, medium, and fast), at three possible angles relative to the horizontal plane (0, 15°, 30°), and in three different manners (constant speed, deceleration, and acceleration), reaching 27 distinct combinations of movement parameters with the intention of moderating the expected learning effect and providing varying levels of difficulty for further fMRI models and analyses. The projectile (diameter 0.3 cm) was launched from the lower right corner of the screen at a constant speed of 20.0 cm/s on a vertical, unchanging trajectory. The subjects were instructed to press the key only once at the optimal time for the projectile to intercept the moving circular target in the upper right corner of the screen. A successful interception was followed by a small explosion animation as feedback for the subject (Figure 1(b)). The participants were specifically asked to visually follow the target at all times and encouraged to use the same strategy throughout the experiment. Due to the varying, complex movement parameters of the target, simplified approaches such as fixating the gaze on a launching area consciously estimated based on the previous trials and waiting for the target to reach the area or mentally counting the time from the target's appearance to a preselected point would prove useless.

The experiment was organized into 6 blocks, each formed by 54 trials, with pseudorandomized combinations of the target movement parameters to provide 12 repetitions of each combination for a total of 324 trials. We employed a counterbalanced presentation of various stimuli types within each block to minimize the repetition of the same movement parameter combinations in consecutive trials. The duration of one trial was on average 3.5 s.

The entire paradigm consisted of a training session (performed while scanning, but not subject to analysis) and the main task. The duration of the whole session (including the acquisition of anatomical scans, the training session, and the main session with short breaks between the blocks to prevent motor or cognitive fatigue) was about 50 minutes. The task was presented using the LabVIEW 6.1 (National Instruments, Austin, TX, USA) interface.

### 2.3. fMRI Data Acquisition

Imaging was performed at the radiology department of St. Anne's University Hospital, Brno, on a 1.5 T Siemens Symphony scanner equipped with Numaris 4 System (MRease). High-resolution anatomical T1-weighted images were acquired using the magnetization-prepared rapid gradient-echo (MPRAGE) sequence (TR = 1.7 s, TE = 3.93 ms, TI = 1100 ms, in-plane voxel size 0.96 × 0.96 mm, 160 sagittal slices, slice thickness 1.17 mm, matrix 256 × 256 × 160). A total of 850 volumes of functional images were acquired during the entire run (TR = 2.3 s, TE = 35 ms, FA = 90°, FOV = 220 × 180 mm, in-plane voxel size = 3.44 × 3.44 mm, 28 axial slices, slice thickness 4.40 mm), but only 490 volumes corresponding to the main task were used for further analyses (the remaining volumes scanned during the training part were discarded). Two dummy scans, acquired before each fMRI run in order for the fMRI signal to reach a steady state, were discarded from the analysis as well.

### 2.4. Analysis of Behavioral Data

The main variable of interest was the success rate (i.e., the hit ratio) across the different types of movements (stable speed, acceleration, and deceleration) and speed types. The angle was excluded from the analyses, since it was proven to have no effect on the hit ratio [20, 38]. We computed the average success rate in each subject for every movement type in order to be able to use parametric statistical analysis. The effects of the movement parameters on the main variable were assessed using ANOVA analysis, with the hit ratio as a dependent variable and the speed and the movement type as independent factors. The analyses were performed using the Statistica 12 software (Statsoft Inc., Oklahoma, USA).

### 2.5. Analysis of fMRI Data

The fMRI data were preprocessed and analyzed using SPM8 (Wellcome Department of Cognitive Neurology, London, UK) implemented in Matlab R2013 (Mathworks Inc., Sherborn, MA, USA). The images were realigned to correct for head movement effects. No subject's movements exceeded 3 mm in each spatial direction or 3° of rotation. Afterwards, the coregistration of functional and anatomical images, interpolation in time to correct for volume acquisition phase progression, spatial normalization into the stereotactic Montreal Neurological Institute (MNI) space, and spatial smoothing using an isotropic Gaussian kernel of 8 mm full-width at half-maximum were performed. To minimize low frequency artifacts possibly associated with the block length, the data were high-pass filtered with a Gaussian kernel filter of 128 s.

At the first level, the general linear model of BOLD activations consisted of bidirectional comparisons of successful hits and errors in each subject separately. The individual design matrix for every participant also included the speed, as interpreting the results of this parameter was more straightforward than with the complicated scheme of the movement type (acceleration, deceleration, and stable speed). The onset was modelled as the moment the green circular target appeared on the left side of the screen and the end was the moment when the subject pressed the key to launch the projectile.

In the analysis of the main hypothesis, six contrast maps were created at the first level (two possible results [hit or miss] × three speeds) and a second level factorial design (2 × 3) was implemented with the following factors: success (two levels: hit and miss) and speed (three levels: slow, medium, and high). The analysis of our secondary aim, the gender distinctions, was based on a first level model (3 speeds × 3 movement types (stable, acceleration, deceleration)) and the successive second level model with gender, speed, and the movement type as factors (i.e., 2 × 3 × 3) and the respective hit ratios for the individual target movement parameters as a nuisance variable to control for the effect associated primarily with the individual success rate differences.

The results were considered significant at *p* < 0.05; family-wise error (FWE) corrected for multiple comparisons at the whole brain level with a cluster threshold of 35 contiguous voxels in all the comparisons.

## 3. Results

### 3.1. Behavioral Results

**Figure 2:**
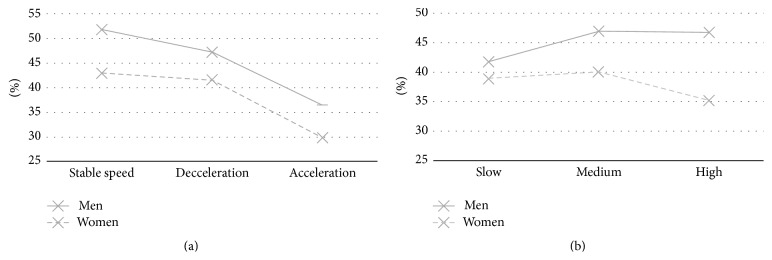
Graphical representation of performance in both genders and the influence of the movement parameters. (a) Mean success rate as a function of acceleration in males and females. (b) Mean success rate as a function of speed in males and females.

The analysis of pooled data provided a mean hit ratio of 0.4281 (SD = 0.1534), with a significant effect of movement type (*F*_2, 354_ = 24.75; *p* < 0.001) (Figure 2(a)) and a borderline trend in the effect of speed (*F*_2, 354_ = 2.054; *p* = 0.11) (Figure 2(b)). Overall, acceleration was associated with a lower success rate than either deceleration or stable speed. Furthermore, the character and timing of errors were put to test, distinguishing between early errors (i.e., errors when the subject pressed the button too early) and late errors (the subject pressed the button too late). There was no difference between the occurrence of early and late errors; early errors constituted 51.6% and the late errors, 48.4% of all the errors (*p* > 0.05). Due to this even distribution of errors, the average response time (period between the onset of the stimulus and the motor response—key press) did not differ between successful trials, “hits” (1.301 s), and unsuccessful trials, “misses” (1.265 s) (*p* > 0.05).

Interestingly, significant gender differences were found, with a mean hit ratio of 0.4589 (SD = 0.1552) in men and 0.3803 (SD = 0.1383) in women (*p* < 0.001). Sex did not interact significantly with acceleration (*F*_2, 354_ = 0.2864; *p* = 0.75) (Figure 2(a)) or speed (only a borderline trend of performance decrease in women with higher speeds than men—*F*_2, 354_ = 2.235; *p* = 0.11) (Figure 2(b)).

### 3.2. Activation Maps

**Figure 3:**
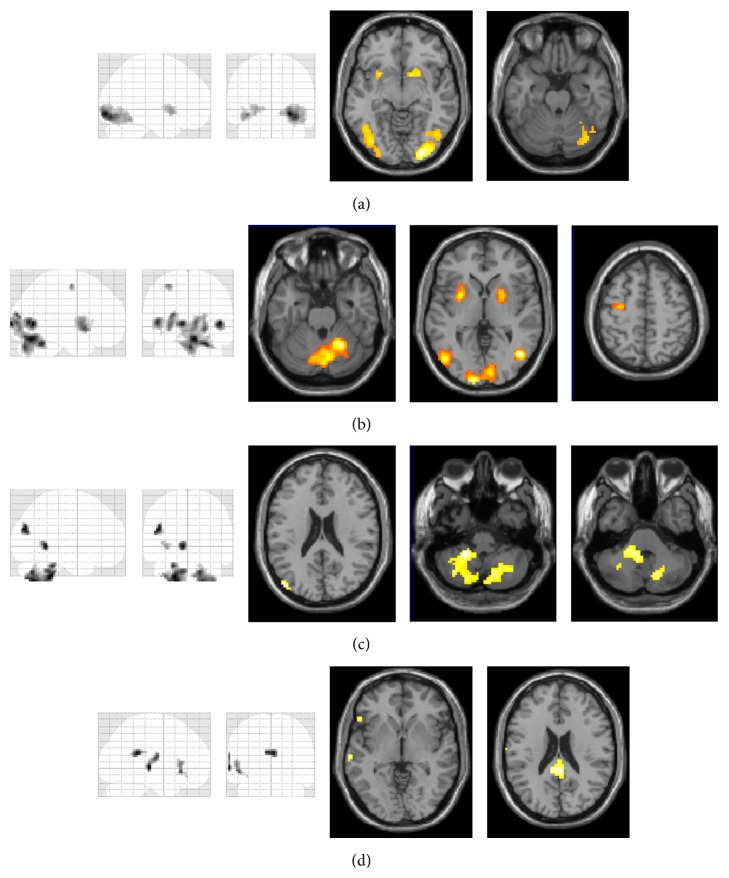
Results of the 3 × 2 ANOVA (*p* < 0.05, FWE-corrected at the whole brain level): (a) main effect hit versus miss (hit-miss) (threshold *T* = 4.56). (b) Main effect of speed (*F*-contrast) (threshold *F* = 13.36). (c) Female > male (threshold *T* = 4.56). (d) Male > female (threshold *T* = 4.56). Radiological conventions for the laterality were used where the right side in the figure corresponds to the right side in the scanned area and vice versa.

The results of the 2 × 3 ANOVA (hit/miss × speed) were as follows:

(1) Main effect of hit versus miss: The areas significantly more activated in successful trials included the basal ganglia (specifically the putamen and caudate bilaterally) and a larger cluster comprising the posterior cerebellum (lobule VI and crus 1 on the right side) and the inferior part of the occipital lobe (Figure 3(a), Table 1). The reverse contrast (miss > hit) did not provide any results surviving the threshold of *p* < 0.05, FWE-corrected.
(2) The effect of speed: Speed changes of the target were closely associated with cerebellar activity in the posterior cerebellum (specifically vermis 6 and vermis 7, bilateral lobules VI, and crura 1, with the predominance of the right cerebellar hemisphere) and, furthermore, with the right middle temporal lobe, the middle occipital lobe on the left side, and smaller clusters of activity in the putamen bilaterally and in the left precentral gyrus (Figure 3(b), Table 1).
(3) Interaction of hit versus miss and speed: No clusters survived at the level of *p* < 0.05 with FWE-correction at the whole brain level.

The analysis of the effect of gender, with the hit ratio as a nuisance variable due to performance differences (see the behavioral results), yielded the following activation maps:

(1) Women > men: Figure 3(c) demonstrates the patterns of hyperactivation in women when compared to men, comprising primarily of the posterior cerebellum (specifically the left lobules IV-V, VIII, IX, and the right VIII, VII, and VI with left-sided predominance). Further regions found in this contrast included two clusters in the left temporal gyrus (Brodmann area 39) (Figure 3(c), Table 1).
(2) Men > women: The reverse contrast revealed differences of lesser magnitude than the ones reported above, including the cingulum and Brodmann areas 45 and 21.
(3) The interaction between gender and speed or movement type did not yield any results surviving the threshold of *p* > 0.0, FWE-corrected at the level of whole brain.

## 4. Discussion

Research efforts have been increasingly directed towards understanding the neuroanatomical substrates of time perception by studying the characteristic patterns of neuronal activity and behavioral correlates in both healthy individuals and specific patient populations. Although a large corpus of research has implicated complex, overlapping networks bringing together information from multiple modalities, including the basal ganglia, cerebellum, posterior parietal cortex, and frontal cortex, substantial disagreement persists regarding the relevance of these structures and the specific computing models used to delineate temporal parameters from various inputs [1, 6, 7, 9, 10]. Functional neuroimaging studies have consistently used tasks based on paced finger tapping and temporal discrimination [3]. Building upon the previous research, but with a substantial step forwards, the current study is not concerned with the simple ability to maintain certain intervals or to assess and compare the length of various stimuli, that is, with retrospective temporal processing. It is designed to analyze the neural networks involved in predictive timing processes in the subsecond spectrum that enable preemptive action on the basis of the estimated future state of our environment, an essential ability whose absence would virtually preclude even simply catching a ball.

Our results highlight the specific functional role of several areas, including the putamen and caudate, the temporal cortex, and primarily the cerebellum, an area previously commonly associated with temporal tasks, although probably of an underspecified level of contribution [11]. Our finding of high activation in the cerebellum associated with increasing speed, and hence also with the difficulty of the task, is well in accordance with previous studies of subsecond spectrum retrospective temporal processing utilizing transcranial magnetic stimulation of the cerebellum [39–41] and imaging methods [25, 42–46], and also with the hypothesis that this neural node acts as a timing system for brief intervals [47]. Even if reported as the most commonly engaged structure in retrospective subsecond temporal computing [5], the extent of the volume of activated cerebellar tissue and the dominance of the cerebellar cluster in the current study far exceeds the volumes and the rank among the other neural nodes reported in retrospective timing tasks [3]. In contrast to the prevailing retrospective tasks, only a few studies have focused primarily on temporal predictions; the anterior-inferior parietal cortex and ventral premotor cortex in the Posner paradigm [48] and the anterior-inferior parietal cortex in trajectory prediction [49] have been suggested. The findings presented here are also very much in keeping with the analysis of perceptual prediction change and trajectory extrapolation associated with the notable engagement of the posterior cerebellum [50].

Turning to the other regions implied in the current study, the putamen, clearly revealed here in both the speed and success versus failure analysis, is extensively documented in temporal processing in the subsecond and multisecond range in both animals and humans (for review see [5]). It was highlighted in tasks requiring the comparison of durations with a probe stimulus [51, 52] and even proposed as a core timer across the whole temporal spectrum [5], responsible for the early stages of temporal computation [51], temporary storage [52], or as a functional center in stimulus encoding, integrating the temporal signals and generating a subjective perception [11]. Our findings of a significant relation of putaminal activity to the hit ratio in predictive temporal computing not only resonate with the previous hypotheses, but also go beyond these and underscore its function as a neural node engaged in the evaluation of success and precision of the undergoing prospective temporal analysis. Furthermore, although often not commented upon, the activity in the middle temporal lobe during timing tasks has also been implicated before [15, 51–53]. This supramodal involvement of the auditory cortex was demonstrated both in auditory [54] and visual timing [55].

Our secondary aim, delineating gender differences in predictive timing, was already partly addressed in the analysis of behavioral data revealing a significantly lower success rate in women. The emergence of the cerebellum as the main hyperactivity cluster in women in the neuroimaging analysis, even when corrected for the hit ratio difference, may seem rather surprising, as the cerebellum is traditionally considered to be a fairly monomorphic structure with minimal sex dissimilarities. Neuroimaging studies seem to provide contradictory results, and a higher volume of cerebellar hemispheres in men [26, 56], analogous to observations of the cerebral cortex [57], was reported, although other analyses, volumetric assessments based on MRI [58] and examination of fixed human tissue [59], failed to reveal a significant relationship between gender and cerebellar structure. The situation is similar in the field of functional studies. PET confusingly revealed increased metabolism in women in the cerebellum [27], corresponding to the findings of our study, decreased metabolism in women in the cerebellum [60], or no gender differences at all in the infratentorial area [61]. The lack of association with gender, though not completely surprising given the character of the stimulation task, was also reported in fMRI studies [62, 63]. On the other hand, strong cerebellar hyperactivity in women during simple silent counting, markedly similar to our results, provided a foundation for the hypothesis of distinct cognitive and executive strategies in response to various stimuli between the genders [64]. Moreover, behavioral performance differences between the genders have been well known for decades, for example, in language, dominated by women, and visuospatial tasks, where men show superior results [28]. Due to the versatile nature of cerebellar functions, this neural node has been implicated in many of those areas [65–67]. Hence, even the extent and character of cerebellar engagement may be of different magnitude in men and women. Future studies incorporating complex analyses will be helpful in expanding upon our current results and confirming the disputable dissimilarities in activity patterns in the infratentorial area. The mechanisms underlying the increased cerebellar activity in women may have the character of a compensatory hyperactivation of one node of a complex neural network commonly responsible for temporal analysis, but without further support from other studies using various methods, this hypothesis is far from elucidating this poorly understood issue.

A number of points need to be considered with our results. First, given the temporal constraints of fMRI, the plausibility of disambiguation of individual components and their specific functional signature in the process of prospective temporal timing in intervals of short duration used in our task may be considered borderline at best. Ergo, no further hypotheses about the probable temporal processing model utilized in predictive timing may be provided, except for the obvious statement of multiple neural nodes engaged, with cerebellar dominance. Second, the emergence of occipital cortical areas in the analysis of hit versus miss and, to a lesser extent, even in the analysis of the speed effect may not be surprising given the increased demands for both primary and secondary image processing necessary for the successful interception of the moving target. However, in the light of this distinction, the interpretation of other significant clusters as core structures for predictive temporal processing might be challenged. Moreover, the notion of variable analyzed time interval with the offset of the regressor corresponding to the motor response may point to possible contamination with the signal associated with motor processing due to the temporal proximity of early motor responses (early errors). However, due to the even distribution of errors between too early and too late responses, this convolution seems unlikely. Nonetheless, our results are well in accord with the previous body of research on retrospective timing, thus allowing us confidence in our findings. The final issue worth reiterating in the context of gender distinctions is the complexity of the task; it consists of series of component processes, and the neural activity in males and females may show differences for only specific segments, unfortunately outside the temporal resolution of fMRI. Further work will be necessary to tease apart the extent to which cerebellar activation varies between genders and to develop experimental tasks optimized to probe specific hypotheses about the gender differences.

Taken together, our findings underscore the pivotal role of the cerebellum in predictive temporal processing, with considerably higher engagement than in retrospective timing tasks, and provide novel insights for understanding different neural mechanisms in men and women. Moreover, our results offer a more nuanced perspective on the contribution of the putamen in analyzing predictive temporal processing precision and success as a possible node in a feedback loop activated more in successful trials than in errors. Despite the increasing amount of research, the complex function of the cerebellum in predictive timing remains notably underexplored. It will be important for future studies to expand upon the current results in other domains of complex prospective timing.

## Figures and Tables

**Table 1:** Anatomical localization of clusters in the activation analysis (threshold of *p* < 0.05, FEW-corrected). Data provided for the main effect of hit versus miss, the effect of speed, and the gender differences.

Anatomical regions	Brodmann area/lobules in cerebellum	Side	Volume (in voxels)	*p* value (FWE)	*T*-score of local max	MNI coordinates of local maxima		
*Hit > miss*								
Occipital lobe	BA 18-19	R	433	<0.001	8.75	27	−91	−8
	BA 18-19	L	174	<0.001	6.68	−39	−76	−11
Putamen, caudate		R	63	<0.001	6.05	21	11	−5
Putamen, caudate		L	80	<0.001	5.81	−24	8	−2
Cerebellum	Lobule VI, crus 1	R	48	<0.001	5.55	36	−76	−20

Anatomical regions	Brodmann area/lobules in cerebellum	Side	Volume (in voxels)	*p* value (FWE)	*F*-score of local max	MNI coordinates of local maxima		

*Main effect of speed*								
Cerebellum	Vermis 6, vermis 7 Lobule VI, crus 1	C BIL	597	<0.001	38.73	6	−70	−20
Middle temporal gyrus	BA 37	R	94	<0.001	36.82	45	−67	1
	BA 37	L	161	<0.001	34.73	−48	−70	4
Occipital lobe	BA 18-19	L	536	<0.001	35.02	−12	−100	1
Putamen		R	144	<0.001	28.72	24	8	−5
Putamen		L	149	<0.001	25.97	−30	5	1
Precentral gyrus	BA 6	L	36	<0.001	24.35	−30	−10	58

Anatomical regions	Brodmann area/lobules in cerebellum	Side	Volume (in voxels)	*p* value (FWE)	*T*-score of local max	MNI coordinates of local maxima		

*Female > male*								
Middle temporal gyrus	BA 39	L	35	<0.001	7.48	−48	−79	22
Cerebellum	Lobule IV-V	L	47	<0.001	7.36	−9	−49	−2
	Lobule VIII, IX, IV-V	L	430	<0.001	7.36	−21	−43	−44
	Lobule VIII, VII, VI	R	227	<0.001	6.76	15	−70	−38
Middle temporal gyrus		L	35	0.001	5.59	−36	−52	−2

*Male > female*								
Middle temporal gyrus	BA 21	L	40	<0.001	7.17	−66	−25	−2
Cingulum		C	83	<0.001	6.95	−6	−40	22
Inferior frontal gyrus	BA 45	L	48	<0.001	5.94	−51	26	−8

BA = Brodmann area, L = left, R = right, C = central, and BIL = bilateral.
